# Optical anisotropy composition of benign and malignant prostate tissues revealed by Mueller-matrix imaging

**DOI:** 10.1364/BOE.464420

**Published:** 2022-10-25

**Authors:** Oleksii Sieryi, Yuriy Ushenko, Volodimir Ushenko, Olexander Dubolazov, Anastasia V. Syvokorovskaya, Oleh Vanchulyak, Alexander G. Ushenko, Mykhailo Gorsky, Yuriy Tomka, Alexander Bykov, Wenjun Yan, Igor Meglinski

**Affiliations:** 1 Optoelectronics and Measurement Techniques, University of Oulu, Oulu, Finland; 2 Optics and Publishing Department, Chernivtsi National University, Chernivtsi, Ukraine; 3 Department of Forensic Medicine and Medical Law, Bukovinian State Medical University, Chernivtsi, Ukraine; 4 College of Electrical Engineering, Taizhou Research Institute, Zhejiang University, Taizhou, China; 5 College of Engineering and Physical Sciences, Aston University, Birmingham, UK

## Abstract

A Mueller matrix imaging approach is employed to disclose the three-dimensional composition framework of optical anisotropy within cancerous biotissues. Visualized by the Mueller matrix technique spatial architecture of optical anisotropy of tissues is characterised by high-order statistical moments. Thus, quantitative analysis of the spatial distribution of optical anisotropy, such as linear and circular birefringence and dichroism, is revealed by using high-order statistical moments, enabling definitively discriminate prostate adenoma and carcinoma. The developed approach provides greater (>90%) accuracy of diagnostic achieved by using either the 3-rd or 4-th order statistical moments of the linear anisotropy parameters. Noticeable difference is observed between prostate adenoma and carcinoma tissue samples in terms of the extinction coefficient and the degree of depolarisation. Juxtaposition to other optical diagnostic modalities demonstrates the greater accuracy of the approach described herein, paving the way for its wider application in cancer diagnosis and tissue characterization.

## 1. Introduction

Prostate cancer (PC) is one of the most common malignant neoplasms leading in terms of growth ( ∼ 1.41 million cases in 2020 [1]) and being second among the causes of death in men [2]). Facile and rapid differentiation of prostate tissues is a critical medical challenge [3]) with faster and more accurate tumour diagnosis allowing accelerated intervention and management, leading to significant improvements in patient outcomes [4,5]). State-of-the-art PC diagnosis is based on a combined use of several methods, including blood test for prostate-specific antigen, palpation of the prostate through the rectum (digital rectal examination), rectal ultrasound and and multi-parametric magnetic resonance imaging (MRI) for local staging [1]. Current clinical practice of PC screening is focused on the quantitative evaluation of several most indicative parameters of metabolic by-products in blood. As a result, clinical PC diagnosis is overburdened with data analysis, associated errors, low specificity, and high cost. In addition, conventional PC diagnostic approaches allow to evaluate morphological changes within the tissues with quite low ( ∼100−200μm ) spatial resolution [2], and does not give an opportunity for the diagnosis at early stage of the disease.

A number of advancements in the PC examination are credited to optical imaging, beginning with visualizing cancer cells by quantitative microscopy [6]. Tissue biopsy with microscopy-based observation of the cancer cells forms the basis of histo-pathology analysis, leading in the systematic understanding of neoplasia origins. The biopsy approach is based on rendering of very tiny ( ∼3−5μm ) slices of tissues stained with appropriate dyes to enhance the visibility contrast and identification of margins of cancerous area. This cancer screening approach is currently the ’gold standard’ in the clinical practice of confirmation of the neoplastic disease and determining whether the neoplasms are benign or malignant, as well as evaluating its aggressiveness. Despite of a wide use in routine day-to-day clinical practice this diagnostic technique is still very subjective and don’t provide the possibility of quantitative characterisation of the parameters of optical images of preparations of benign and malignant tumors.

Polarisation-based imaging has been successfully applied in the field of cancer diagnosis [7,8]. The morphological alterations in tumor micro-environment comprising the stroma, mostly contained from collagen, become differ from the healthy tissue morphology, and can be observed and assessed quantitatively by polarization imaging techniques. This provides and opportunity to demarcate the healthy and cancerous tissues, detect precancerous lesions, as well as to examine various aspects of tumors development including metastasis tracing. The peculiarities of numerous applications of optical polarization methods in tissue characterization, early cancer diagnosis and monitoring of treatment efficiency are widely reported elsewhere [7,9–20]. The differential Muller-matrix (MM) polarimetry reconstructing spatial distributions of optical anisotropy within cancer tissues, provide a new diagnostic approach to explore pathological conditions in biological tissues [21–29]. The 3D imaging of cancerous tissues with polarization-sensitive optical coherence tomography (PS-OCT) allows quite accurate ( ∼8μm−10μm spatial resolution) screening of thickening or scarring of tissues, as well as demarcating tumors areas with low fibrosis [30]. A comparative analysis of modern methods of clinical cancer diagnosis reveal a number of unsolved issues:

• complexity of ’interdisciplinary’ diagnosis of living biological systems utilizing principles of optical imaging, polarimetry, laser interferometry and tomography;
• time-consuming multi-parameter analysis of 2D/3D/4D medical images, even with the use of smart machine learning (ML) and deep learning (DL) algorithms and elements of artificial intelligence (AI);
• standardization of a single multi-functional laser polarization-interference biophysical technology for diagnosis and differentiation of prostate cancer stages.

Further development of polarization-based diagnostic modalities based on MM formalism can be combined with modern laser-holographic techniques. Thus, MM differential diagnosis of benign and malignant tumors of human organs (prostate, uterus) was introduced [31,32]. In this approach the polarization-interference 3D layer-by-layer mapping of the initial MM imaging of the optically anisotropic structure of partially depolarizing layers of cancer tissues is implemented. In frame of these studies a direct relationship between a set of statistical moments of the 1-st – 4-th orders, characterizing MM images, and the definitive diagnostic features of the morphological structure of fibrillar and parenchymal biological tissues was established. Whereas, utilizing a statistical analysis of the obtained layered MM images and their derivatives – depolarization maps the accuracy of cancerous tissues demarcation was assessed. While the obtained depolarization maps do not carry direct information about the optical anisotropy of cancerous tissues, the development of this technique was the digital computational reconstruction of optical anisotropy maps of myocardial fibrillar networks and their successful use to differentiate the degree of necrotic changes [33]. The developed approach is using the differential components of the Müller matrices Ossikowski - Devlaminck [21–25] that provides an opportunity for reconstruction of layered maps of average values of linear and circular birefringence and dichroism of partially depolarizing layers of biological tissues with another type of pathology - cancerous tissues. Thus, we expect to expand the functionality of 3D MM layered tomography and obtain new diagnostic markers that will provide high sensitivity and accuracy of differential diagnosis of diffuse histological sections of prostate cancer biopsy.

## 2. Methods and materials

The measurements and image reconstruction of optical anisotropy of cancerous tissues are based on the hybrid use of differential MM imaging and polarization-based layer-by-layer interference approaches, providing, respectively, spatial distribution of optical anisotropy of partially depolarized light and mapping of the field within the layers.

### 2.1 Differential MM imaging approach

The polarization changes within the tissue-like scattering medium is defined by six different optical anisotropic parameters [34]. These parameters correspond to the actual measurements of optical activity, circular dichroism, and four parameters for linear birefringence (two) and linear dichroism (two). Studying by C.R.  Jones in terms of a ’layered’ medium by means of a differential equation analysis, leads to a exponential representation of the Jones matrix. Later R.M.A. Azzam [35] developed and analogous infinitesimal calculus based on MM that can be used for the same purposes. The differential matrix calculus, introduced by Jones, is used to describe the continuous propagation of partially polarized light through linear anisotropic media. Later this theoretical approach was generalized on the basis of analytical decomposition of the total and ’depolarized’ (2-nd order differential matrix) components [21–25].

Thereby, the 1-st order differential matrix is presented in the form of six non-zero elements that carry separate information about the distribution of average (⟨◻⟩) values of linear and circular birefringence (phase anisotropy $\Phi_{0.90},\Phi_{45,135},\Phi_{⊗,⊕}$ and linear and circular dichroism (also known as diattenuation [9,10]) – amplitude anisotropy $\Delta_{0,90},\Delta_{45,135},\Delta_{⊗,⊕}$ .

(1) $$\langle M\rangle =\left\|\begin{array}{cccc} 0 & \left\langle m_{12}\right\rangle & \left\langle m_{13}\right\rangle & \left\langle m_{14}\right\rangle \\ \left\langle m_{21}\right\rangle & 0 & \left\langle m_{23}\right\rangle & \left\langle m_{24}\right\rangle \\ \left\langle m_{31}\right\rangle & \left\langle m_{32}\right\rangle & 0 & \left\langle m_{34}\right\rangle \\ \left\langle m_{41}\right\rangle & \left\langle m_{42}\right\rangle & \left\langle m_{43}\right\rangle & 0 \end{array}\right\|=\left\|\begin{array}{cccc} 0 & \left\langle \Delta_{0,90}\right\rangle & \left\langle \Delta_{45,135}\right\rangle & \left\langle \Delta_{⊗,⊕}\right\rangle \\ \left\langle \Delta_{0,90}\right\rangle & 0 & \left\langle \Phi_{⊗,⊕}\right\rangle & \left\langle -\Phi_{45,135}\right\rangle \\ \left\langle \Delta_{45,135}\right\rangle & \left\langle -\Phi_{⊗,⊕}\right\rangle & 0 & \left\langle \Phi_{0,90}\right\rangle \\ \langle \Delta_{⊗,⊕\rangle } & \left\langle \Phi_{45,135}\right\rangle & \left\langle -\Phi_{0,90}\right\rangle & 0 \end{array}\right\|$$

Here, $\left\langle \Delta_{0,90}\rangle ,\langle \Delta_{45,135}\right\rangle$ are the average values of linear dichroism between the orthogonal components at $0^{\circ }\&90^{\circ }$ and $45^{\circ }\&135^{\circ }$ , respectively; $\left\langle \Phi_{0,90}\rangle ,\langle \Phi_{45,135}\right\rangle$ are the average values of linear birefringence between the orthogonal components at $0^{\circ }\&90^{\circ }$ and $45^{\circ }\&135^{\circ }$ , respectively; $\left\langle \Delta_{⊗,⊕}\rangle ,\langle \Phi_{⊗,⊕}\right\rangle$ are the average values of circular dichroism and birefringence between the right- (⊗) and left- (⊕) circularly polarised components.

The parameters of optical anisotropy are defined as [33]:

(2) $$\langle \Phi_{0,90}\rangle =\frac{2\pi }{\lambda }\Delta n_{0,90}l;\;\Delta n_{0,90}=n_{0}-n_{90};$$

(3) $$\langle \Phi_{45,135}\rangle =\frac{2\pi }{\lambda }\Delta n_{45,135}l;\;\Delta n_{45,135}=n_{45}-n_{135};$$

(4) $$\langle \Phi_{⊗,⊕}\rangle =\frac{2\pi }{\lambda }\Delta n_{⊗,⊕}l;\;\Delta n_{⊗,⊕}=n_{⊗}-n_{⊕;}$$

(5) $$\langle \Delta_{0,90}\rangle =\frac{2\pi }{\lambda }\Delta \tau_{0,90}l;\;\Delta \tau_{0,90}=\tau_{0}-\tau_{90};$$

(6) $$\langle \Delta_{45,135}\rangle =\frac{2\pi }{\lambda }\Delta \tau_{45,135}l;\;\Delta \tau_{45,135}=\tau_{45}-\tau_{135};$$

(7) $$\langle \Delta_{⊗,⊕}\rangle =\frac{2\pi }{\lambda }\Delta \tau_{⊗,⊕}l;\;\Delta \tau_{⊗,⊕}=\tau_{⊗}-\tau_{⊕}.$$

Here, $n_{j}$ and $\tau_{j}$ are the refractive index and absorption coefficient for the j -polarised component (where j defines orientation of the orthogonal components $0^{\circ },90^{\circ },45^{\circ },135^{\circ },⊗\;or\;⊕)$ of the incident laser light, λ is the wavelength of incident laser light, l is the thickness of sample through which the light propagates.

Analytical relationship was found between the elements $\left\langle m_{ik}\right\rangle$ of the 1-st order differential matrix ⟨M⟩ and the experimentally measured elements $f_{ik}$ of the full MM {F} for a sample of tissue partially depolarizing light [36]:

(8) $$\langle M\rangle =0.5l^{-1}\left\|\begin{array}{cccc} 0 & ln(f_{12}f_{21}) & ln(f_{13}f_{31}) & ln(f_{14}f_{41})\\ ln(f_{12}f_{21}) & 0 & ln\left(\frac{f_{23}}{f_{32}}\right) & ln\left(\frac{f_{24}}{f_{42}}\right)\\ ln(f_{13}f_{31}) & ln\left(\frac{f_{32}}{f_{23}}\right) & 0 & ln\left(\frac{f_{34}}{f_{43}}\right)\\ ln(f_{14}f_{41}) & ln\left(\frac{f_{42}}{f_{24}}\right) & ln\left(\frac{f_{43}}{f_{34}}\right) & 0 \end{array}\right\|.$$

Taking into combined consideration (2)–(7) and (8) allows one to derive expression for the reconstruction of average values of the six phase parameters:

(9) $$\langle \Phi_{0,90}\rangle =ln\left(\frac{f_{24}}{f_{42}}\right)$$

(10) $$\langle \Phi_{45,135}\rangle =ln\left(\frac{f_{34}}{f_{43}}\right)$$

(11) $$\langle \Phi_{⊗,⊕}\rangle =ln\left(\frac{f_{32}}{f_{23}}\right),$$

and amplitude anisotropy:

(12) $$\langle \Delta_{0,90}\rangle =ln(f_{12}f_{21});$$

(13) $$\langle \Delta_{45,135}\rangle =ln(f_{13}f_{31});$$

(14) $$\langle \Delta_{⊗,⊕\rangle }=ln(f_{14}f_{41}).$$

To describe linear birefringence and dichroism we use the generalized parameters of the linear birefringence $\left(\Phi_{L}\right)$ and linear dichroism $\left(\Delta_{L}\right)$ [37]:

(15) $$\langle \Phi_{L}\rangle =\sqrt{{\left(ln\left(\frac{f_{24}}{f_{42}}\right)\right)}^{2}+{\left(ln\left(\frac{f_{34}}{f_{32}}\right)\right)}^{2}}$$

(16) $$\langle \Delta_{L}\rangle =\sqrt{ln{\left(f_{12}f_{21}\right)}^{2}+ln{\left(f_{13}f_{31}\right)}^{2}}$$

### 2.2 Polarization-based interference approach

The use of complex polarization-interference and digital holographic reconstruction of layer-by-layer spatial distribution of the fields of complex amplitudes induced by optical anisotropy of biological tissues at different depths is well known for a while [38–40]. We adopted the layered tomography mapping of optical anisotropy of the histological sections of miocard [33]. Briefly, the reconstruction of spatial layered distribution of the optical anisotropy is based on the following steps:

(i) The polarizers are used to get six distinct polarization states in both the sampling ( Ir ) and reference ( Re ) beams: {Ir−Re}: $0^{\circ }$ ; $90^{\circ }$ ; $45^{\circ }$ ; $135^{\circ }$ ; ⊗ ; ⊕ . Wherein, the difference in optical path-lengths (Δl) of reference and sampling beams of the polarized interferometer do not exceed the coherence length ( L ) of laser ( Δl=15cm and L=30cm ).
(ii) The registration of two partial interference patterns is performed through the polarizer-analyzer with the orientation of the transmission plane at angles $\Omega =0^{\circ }$ ; $\Omega =90^{\circ }$ .
(iii) Two-dimensional discrete Fourier transform W(υ,ν) is applied to the images of partial interference distribution. The W(υ,ν) of a two-dimensional array $I_{\Omega =0^{\circ };90^{\circ }}(x,y)$ – a function of two discrete variables coordinates (x,y) is defined as [41]:
(17) $$\begin{array}{ll} & W_{\Omega =0^{\circ };90^{\circ }}^{0^{\circ };90^{\circ };45^{\circ };135^{\circ };⊗;⊕}(\upsilon ,\nu )=\\ & =\frac{1}{A\times B}\sum_{a=0}^{A-1}\sum_{b=0}^{B-1}I_{\Omega =0^{\circ };90^{\circ }}^{0^{\circ };90^{\circ };45^{\circ };135^{\circ };⊗;⊕}(a,b)\exp\left[-i2\pi \left(\frac{a\times \upsilon }{A}+\frac{b\times \nu }{B}\right)\right], \end{array}$$
  where $I_{\Omega =0^{\circ };90^{\circ }}^{0^{\circ };90^{\circ };45^{\circ };135^{\circ };⊗;⊕}(a,b)=E_{\Omega =0^{\circ };90^{\circ }}^{0^{\circ };90^{\circ };45^{\circ };135^{\circ };⊗;⊕}(a,b){E_{◻[a]}^{*}}_{\Omega =0^{\circ };90^{\circ }}^{0^{\circ };90^{\circ };45^{\circ };135^{\circ };⊗;⊕}(a,b)$ are the spatial distributions of the intensity of interference pattern treated by the analyser orientated to its transmission axis at the angles $\Omega =0^{\circ }$ ; $\Omega =90^{\circ }$ ; $E_{\Omega =0^{\circ };90^{\circ }}^{0^{\circ };90^{\circ };45^{\circ };135^{\circ };⊗;⊕}(a,b)$ are the orthogonal projections of the complex amplitudes; ∗ denotes the complex conjugation operation; (υ,ν) are the spatial frequencies in the x and y directions respectively; and (A,B) are the number of pixels of the CCD camera in the a and b directions respectively, such that 0≤a,υ≤A and 0≤b,ν≤B .
(iv) One subsequently obtains (for each state of polarization Ir−Re ) a distribution of complex amplitudes:
(18) $$\left\{ \begin{array}{l} \Omega_{0^{\circ }}\to \left|E_{\Omega =0^{\circ }}^{0^{\circ };90^{\circ };45^{\circ };135^{\circ };⊗;⊕}\right|;\\ \Omega_{90^{\circ }}\to \left|E_{\Omega =0^{\circ }}^{0^{\circ };90^{\circ };45^{\circ };135^{\circ };⊗;⊕}\right|\exp\left(i(\varphi_{90^{\circ }}-\varphi_{0^{\circ }})\right) \end{array}\right.$$
  in different phase planes $\varphi_{k}=(\varphi_{90^{\circ }}-\varphi_{0^{\circ }})$ of the object field, separated by an arbitrary step of Δφ .
(v) In each phase plane $\varphi_{k}$ the corresponding sets of parameters of the Stokes vector and polarization parameters of the object field of the biological layer are calculated:
(19) $$\left\{ \begin{array}{l} S_{1}^{0^{\circ };90^{\circ };45^{\circ };135^{\circ };⊗;⊕}(\varphi_{k},a,b)=(|E_{0}{|}^{2}+|E_{90}{|}^{2})(\varphi_{k},a,b);\\ S_{2}^{0^{\circ };90^{\circ };45^{\circ };135^{\circ };⊗;⊕}(\varphi_{k},a,b)=(|E_{0}{|}^{2}-|E_{90}{|}^{2})(\varphi_{k},a,b);\\ S_{3}^{0^{\circ };90^{\circ };45^{\circ };135^{\circ };⊗;⊕}(\varphi_{k},a,b)=2Re|E_{0}E_{90}^{*}|(\varphi_{k},a,b);\\ S_{4}^{0^{\circ };90^{\circ };45^{\circ };135^{\circ };⊗;⊕}(\varphi_{k},a,b)=2Im|E_{0}E_{90}^{*}|(\varphi_{k},a,b). \end{array}\right.$$

Based on relations (17)–(19), the set of elements of the MM {F} is calculated using the following Stokes-polarimetric relations [39,40]:

(20) $$\begin{array}{ll} \{F\}(\varphi_{k}, & a,b)=\left\|\begin{array}{cccc} f_{11} & f_{12} & f_{13} & f_{14}\\ f_{21} & f_{22} & f_{23} & f_{24}\\ f_{31} & f_{23} & f_{24} & f_{34}\\ f_{41} & f_{24} & f_{34} & f_{44} \end{array}\right\|(\varphi_{k},a,b)=\\ & =0.5\left\|\begin{array}{cccc} \left(S_{1}^{0}+S_{1}^{90}\right); & \left(S_{1}^{0}-S_{1}^{90}\right); & \left(S_{1}^{45}-S_{1}^{135}\right); & \left(S_{1}^{⊗}-S_{1}^{⊕}\right);\\ \left(S_{2}^{0}+S_{2}^{90}\right); & \left(S_{2}^{0}-S_{2}^{90}\right); & \left(S_{2}^{45}-S_{2}^{135}\right); & \left(S_{2}^{⊗}-S_{2}^{⊕}\right);\\ \left(S_{3}^{0}+S_{3}^{90}\right); & \left(S_{3}^{0}-S_{3}^{90}\right); & \left(S_{3}^{45}-S_{3}^{135}\right); & \left(S_{3}^{⊗}-S_{3}^{⊕}\right);\\ \left(S_{4}^{0}+S_{4}^{90}\right); & \left(S_{4}^{0}-S_{4}^{90}\right); & \left(S_{4}^{45}-S_{4}^{135}\right); & \left(S_{4}^{⊗}-S_{4}^{⊕}\right). \end{array}\right\|(\varphi_{k},a,b) \end{array}$$

Using the set of distributions (20) and using algorithms (9) – (14), a series of layer-by-layer distributions of the mean values of linear $\left(\langle \Phi_{L}\rangle ,\langle \Delta_{L}\rangle \right)$ and circular $\left(\langle \Phi_{⊗;⊕}\rangle ,\langle \Delta_{⊗;⊕}\rangle \right)$ birefringence and dichroism is obtained

(21) $$\langle \Phi_{L}\rangle (\varphi_{k},a,b)=\sqrt{{\left(ln\left(\frac{\left(ST_{3}^{⊗}-ST_{3}^{⊕}\right)}{\left(ST_{4}^{45}-ST_{4}^{135}\right)}\right)\right)}^{2}+{\left(ln\left(\frac{\left(ST_{3}^{⊗}-ST_{3}^{⊕}\right)}{\left(ST_{4}^{45}-ST_{4}^{135}\right)}\right)\right)}^{2}}(\varphi_{k},a,b);$$

(22) $$\begin{array}{ll} & \langle \Delta_{L}\rangle (\varphi_{k},a,b)=\\ & =\sqrt{{\left(ln\left((ST_{1}^{0}-ST_{1}^{90})(ST_{2}^{0}+ST_{2}^{90})\right)\right)}^{2}+{\left(ln\left((ST_{1}^{45}-ST_{1}^{135})(ST_{3}^{0}+ST_{3}^{90})\right)\right)}^{2}}; \end{array}$$

(23) $$\langle \Phi_{⊗;⊕}\rangle (\varphi_{k},a,b)=ln\left(\frac{\left(ST_{2}^{45}-ST_{2}^{135}\right)}{\left(ST_{3}^{0}-ST_{3}^{90}\right)}\right)(\varphi_{k},a,b);$$

(24) $$\langle \Delta_{⊗;⊕}\rangle (\varphi_{k},a,b)=ln\left(\left(ST_{1}^{⊗}-ST_{1}^{⊕}\right)\left(ST_{4}^{0}+ST_{4}^{90}\right)\right)(\varphi_{k},a,b);$$

### 2.3 3D MM polarization-based imaging

**Fig. 1.**
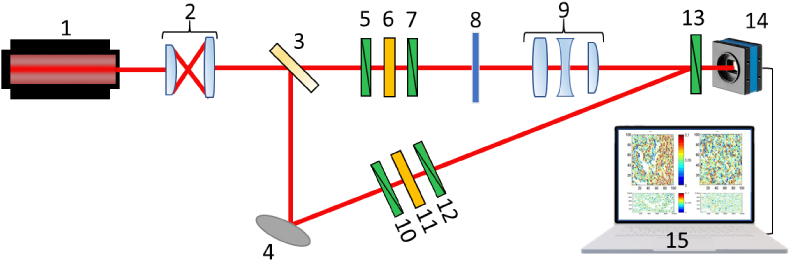
Schematic presentation the experimental 3D MM polarimeter system used in the study. Here: 1 – He-Ne laser; 2 – collimator; 3 – beam splitter; 4 – rotary mirror; 5,7,10,12,13 – polarisation filters; 6,11 – quarter wave plate; 8 – sample under investigation; 9 – strain-free objective; 14 – CCD camera; 15 – PC.

Figure 1 presents the optical arrangement of 3D MM polarimeter experimental system developed in-house [33].

The collimated laser beam $\left(\emptyset =2\times 10^{3}\mu m,\lambda =0.6328\;\mu m\right)$ laser beam is splitted on two equal parts: ’sampling’ and ’reference’ (see Fig. 1). Being transmitted through the polarization filters (5-7, see Fig. 1) the ’sampling’ beam illuminates the tissue sample 8 and passes the image plane of the objective 9 (Nikon CFI Achromat P, focal length 30mm , numerical aperture 0.1, magnification 4× ). The ’reference’ beam, utilizing mirror 4, has been passed through the similar polarization filters (10-12, see Fig. 1). The resulting images of azimuth and ellipticity distributions of tissue sample are transferred to the photosensitive pad of the digital camera 14 (the Imaging Source DMK 41AU02.AS, monochrome 1/2 "CCD, Sony ICX205AL (progressive scan), resolution - 1280×960 , the size of the light-sensitive pad - 7600×6200μm , sensitivity - 0.05 lx, dynamic range - 8 bit, SNR - 9 bit; see Fig. 1). During the measurements the polarization state modulators are operating as:

(i) The transmission axes of polarizers 5 and 10 (see Fig. 1) are oriented perpendicular to the plane of incidence of light. As a result, two collinear polarized laser beams are formed both in ’sampling’ and ’reference’ arms of the interferometer.
(ii) The highest velocity axes of quarter-wave plates 6 and 11 are oriented to each other at the angle of $45^{\circ }$ , relatively to the plane of incidence. Thus, the resulting states of polarization of both ’sampling’ and ’reference’ beams are transformed to the right circular.
(iii) Once the transmission axes of polarizers 7 and 12 (see Fig. 1) are sequentially oriented at the angles $0^{\circ }$ , $45^{\circ }$ , $90^{\circ }$ , $135^{\circ }$ with respect to the plane of incidence, the ’sampling’ and ’reference’ beams are plane-polarized with same intensity.
(iv) By removing the polarizers 7 and 12 (see Fig. 1) the right and left circular polarization in both arms of the interferometer is appeared, whereas the maximum velocity axes of quarter-wave plates 6 and 11 (see Fig. 1) are successively oriented at the angles $45^{\circ }$ and $135^{\circ }$ with respect to the plane of incidence.

The full MM are measured for calibration of the experimental system presented in Fig. 1 and an assessment of measurements error for:

1. ”air” - $\left\|\begin{array}{llll} 1.00 & 0.0092 & 0.0094 & 0.0198\\ 0.0093 & 0.991 & 0.092 & 0.0197\\ 0.0094 & 0.091 & 0.992 & 0.0197\\ 0.0197 & 0.0199 & 0.02 & 0.981 \end{array}\right\|$
2. ”quarter wave-plate” - $\left\|\begin{array}{llll} 1.00 & 0.0092 & 0.0094 & 0.0198\\ 0.0093 & 0.0094 & 0.092 & 0.981\\ 0.0094 & 0.091 & 0.992 & 0.0197\\ 0.0197 & -0.981 & 0.02 & 0.0199 \end{array}\right\|.$

The measurement error of MM elements $f_{i=1;2;3;k=1;2;3}$ did not exceed 1% , $f_{i=1;2;3;4;k=4}$ and $f_{i=4;k=1;2;3;4}$ 2% .

### 2.4 Higher-order statistical analysis

The spatial distribution of optical anisotropy of cancerous tissues are obtained utilizing statistical analysis similar to the one developed earlier [42–45]. This method is based on the calculation of a set of statistical moments of the 1-st - 4-th orders, which characterize the layer-by-layer distributions of the average value of linear and circular birefringence and dichroism – $OA(\varphi_{k},a,b)$ :

(25) $$\begin{array}{r} Z_{1}=\frac{1}{P}\sum_{j=1}^{P}OA{\left(\varphi_{k},a,b\right)}_{j};\\ Z_{2}=\sqrt{\frac{1}{P}\sum_{j=1}^{P}{\left(OA^{2}(\varphi_{k},a,b)\right)}_{j}};\\ Z_{3}=\frac{1}{Z_{2}^{3}}\frac{1}{P}\sum_{j=1}^{P}{\left(OA^{3}(\varphi_{k},a,b)\right)}_{j};\\ Z_{4}=\frac{1}{Z_{2}^{4}}\frac{1}{P}\sum_{j=1}^{P}{\left(OA^{4}(\varphi_{k},a,b)\right)}_{j}, \end{array}$$

where P=A×B are the number of pixels at the camera.

This approach is well suited for statistical analyses of the maps of polarization azimuth and ellipticity, Stokes vector parameters, and MM elements of biological layers partially depolarizing the light, that differ significantly from the Gaussian or normal distribution. In addition to the 1-st and 2-nd order statistical moments $\left(Z_{1;2}\right)$ it’s quite essential to take into account the statistical moments of higher orders $\left(Z_{3;4}\right)$ , which are used to estimate the deviation of polarization distributions from the normal law. Statistical moments of the 3-rd and 4-th orders are used to characterize the asymmetry $\left(Z_{3}\right)$ and the kurtosis $\left(Z_{4}\right)$ of such distributions and their dynamic variations.

Comparative analysis of (25) shows that for the value of the anisotropy parameter $OA∼10^{-3}$ , the statistical moments of higher orders $Z_{3}$ and $Z_{4}$ are significantly (up to three orders of magnitude) larger compared to $Z_{1;2}$ . I.e. even a weak variation in the optical anisotropy of biological tissues is associated with the considerable changes in the statistical distributions $Z_{3;4}$ . Thus, the very high accuracy in definitive differential diagnosis of cancerous and necrotic malformations within the tissue samples can be achieved [46,47]. Taking into account that statistical moments of higher orders are extremely promising for quantitative analysis of complex maps of the parameters of polarization, in current study we are focusing on the screening of benign and malignant prostate tissue samples *in vitro*.

### 2.5 Tissue samples

Table 1 presents the optical and geometric properties of the tissue samples of two groups.

**Table 1.** Optical and geometric properties of native histological sections of prostate tumour biopsies.

Parameter	Group 1	Group 2
Thickness( h,μm )	40±0.45	40±0.45
Attenuation (extinction) coefficient $\tau ,cm^{-1}$	0.85±0.041	0.79±0.037
Degree of depolarisation (Λ,%)	43±0.38	48±0.37

The actual thickness (h,μm) of histological sections of prostate tissue sample was defined by the standard approach utilizing the freezing microtome scale. The extinction coefficient $\left(\tau ,cm^{-1}\right)$ of the prostate tissue samples is assessed by standard photometry measuring attenuation of light propagated through the tissue sample [48], utilizing an integrating sphere [49]. The degree of depolarization (Λ,%) of the histological sections of the samples of prostate tissue carried out by the standard MM polarimetry measurements [46,47]. The statistical significance for a representative set of the number of tissue samples assessed according to the cross-validation method [41]. The standard deviation $\sigma^{2}$ obtained for the statistical moments $Z_{i=1;2;3;4}(n)$ characterises the distribution variations of local contrast maps W(m×n) . The certain number (36 for each group) of samples provided the level $\sigma^{2}\leq 0.025$ . This standard deviation corresponds to a confidence interval p≺0.05 , which demonstrates the statistical reliability of the polarization-interference mapping approach.

## 3. Results and discussion

**Fig. 2.**
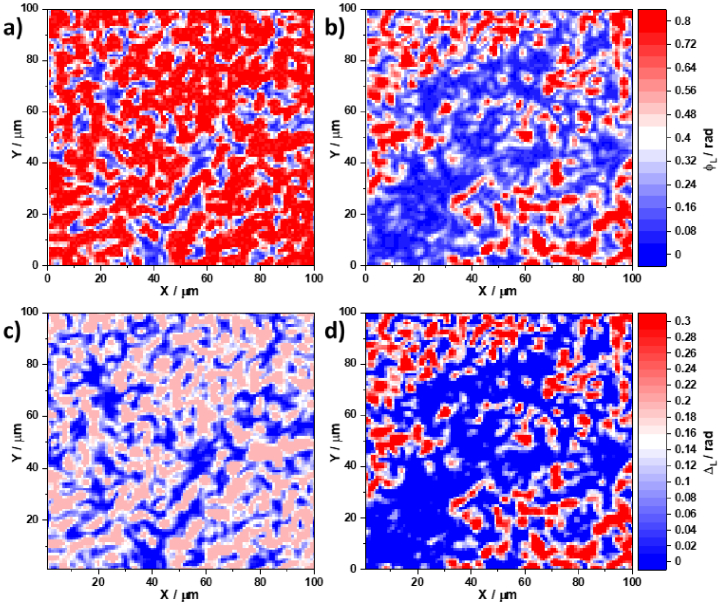
Spatial distribution of linear birefringence $\left\langle \Phi_{L}\right\rangle$ of an adenoma histological section (a); linear birefringence $\left\langle \Phi_{L}\right\rangle$ of a carcinoma histological section (b); linear dichroism $\left\langle \Delta_{L}\right\rangle$ of an adenoma histological section histological section (c); and linear dichroism $\left\langle \Delta_{L}\right\rangle$ of a carcinoma histological section histological section (d).

The differentiating histological biopsy sections of benign and malignant prostate tissues were examined by the 3D MM image reconstruction approach described above. Two groups of histological sections were tested: Benign tissues (adenoma - 26 samples, 0.79≺τ≺0.85 , 43%≺Λ≺48% ); and malignant tissues (carcinoma - 26 samples, 0.81≺τ≺0.84 , 45%≺Λ≺47% ). Figure 2 shows spatial distributions of the linear phase anisotropy (see Fig. 2(a) and 2(b)) and linear amplitude anisotropy (see Fig. 2(c) and 2(d)) parameters of adenoma (see Fig. 2(a) and 2(c)) and carcinoma (see Fig. 2(b) and 2(d)) histological sections, respectively in the $\varphi^{*}=0.6\;\;rad$ phase plane.

**Fig. 3.**
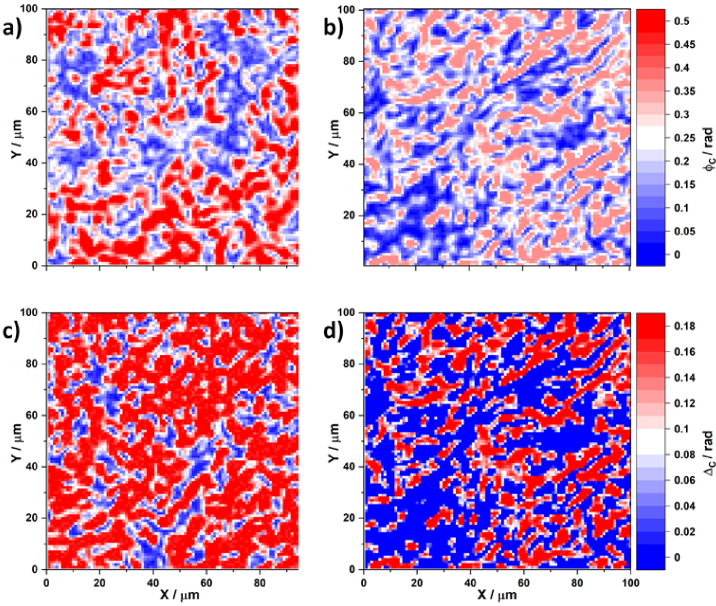
Spatial distribution of circular birefringence $\left\langle \Phi_{⊗,⊕}\right\rangle$ for an adenoma histological section (a); circular birefringence $\left\langle \Phi_{⊗,⊕}\right\rangle$ for a carcinoma histological section (b); circular dichroism $\left\langle \Delta_{⊗,⊕}\right\rangle$ for an adenoma histological section histological section (c); and circular dichroism $\left\langle \Delta_{⊗,⊕}\right\rangle$ for a carcinoma histological section histological section (d).

In a similar manner Fig. 3 presents distributions of circular birefringence and dichroism obtained for adenoma and carcinoma histological sections.

From a physical point of view, previous studies [44–47] have shown that linear birefringence and dichroism are prevalent for adenoma tissues due to the intensive growth of newly formed fibrillar networks. However, the destruction of such structures occurs in a malignant carcinoma state and a corresponding decrease is observed in the phase and amplitude anisotropy owing to spatially-oriented protein networks. These scenarios are clearly illustrated by the obtained maps of distributions $\left\langle \Phi_{L}\rangle (\varphi^{*},x,y\right)$ (see Fig. 2(a) and 2(b)) and $\left\langle \Delta_{L}\rangle (\varphi^{*},x,y\right)$ (see Fig. 2(c) and 2(d)). A significant decrease in both the linear birefringence and dichroism is visible. It appears that the topographic structure observed in the maps of the linear birefringence and dichroism of the histological prostate carcinoma section forms smaller-scale domains when going from a benign to a malignant state. The circular birefringence $\left\langle \Phi_{⊗,⊕)}\rangle (\varphi^{*},x,y\right)$ (see Fig. 3(a) and 3(b)) and the corresponding circular dichroism $\left\langle \Delta_{⊗,⊕}(\varphi^{*},x,y\right)$ (see Fig. 3(c) and 3(d)), determined by the concentration of optically active protein molecules, remain significant for both states. No immediately obvious differences are observable.

**Fig. 4.**
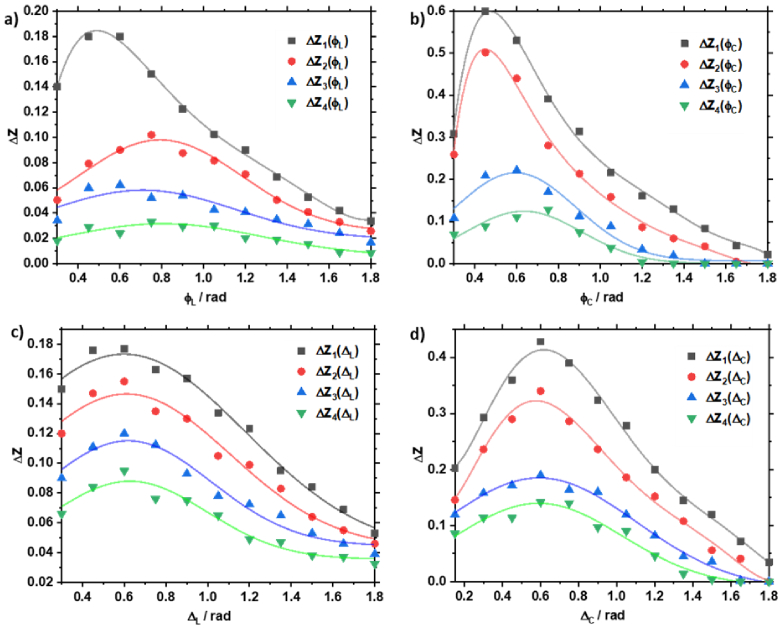
Layered distribution of the optical anisotropy: (a) $\left\langle \Phi_{L}\right\rangle$ , (b) $\left\langle \Phi_{,⊕}\right\rangle$ , (c) $\left\langle \Delta_{L}\right\rangle$ , (d) $\left\langle \Delta_{⊗,⊕}\right\rangle$ of adenoma and prostate carcinoma samples.

Quantitatively, the differences observed between the polycrystalline structures of the histological sections of the biopsy of the different prostate tissues are illustrated by the series of statistical moment dependencies $\Delta Z_{n=1;2;3;4}\left(\varphi_{j};\langle \Phi_{L}\rangle ,\langle \Phi_{⊗,⊕}\rangle ,\langle \Delta_{L}\rangle ,\langle \Delta_{⊗,⊕}\rangle \right)$ shown in Fig. 4.

**Fig. 5.**
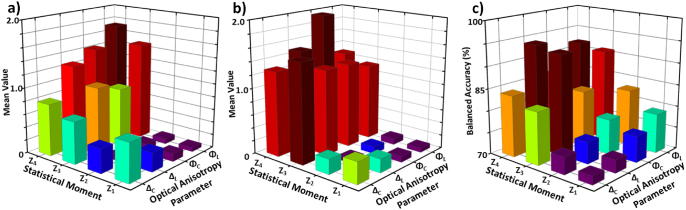
(a) The mean value of the 1-st to 4-th order statistical moments, for each of the four optical anisotropy parameters, for the adenoma sample. (b) The mean value of the first to fourth order statistical moments, for each of the four optical anisotropy parameters, for the carcinoma sample. (c) The balanced accuracy of tumour diagnosis using each combination of statistical moment and optical anisotropy parameters, respectively.

Considering a wider range of phase planes, a comparative analysis of the obtained data found the maximum differences between them in the range 0.3rad≤φ≤0.9rad where there is insignificant scattering multiplicity (i.e., photons on average experience one or fewer scattering events). In the $\varphi^{*}=0.6rad$ phase plane, the means $\Delta {\bar{Z}}_{n=1;2;3;4}^{*}$ and standard deviations $\sigma (\Delta Z_{n}^{*})$ are determined across the entire histological section. Figure 5(a) shows the variation of the mean values of the first to fourth order statistical moments, which characterize the distribution of the four parameters of phase and amplitude anisotropy in the plane $\varphi^{*}=0.6rad$ , for the adenoma sample. Figure 5(b) shows the same, but for the carcinoma sample.

To determine the clinical applicability of this method [50–52] the sensitivity $\left(Se=\frac{a}{a+b}100\%\right)$ , specificity $\left(Sp=\frac{c}{c+d}100\%\right)$ , and balanced accuracy $\left(Ac=\frac{\left(Se+Sp\right)}{2}\right)$ were calculated for each of the statistical moments, $Z_{n=1;2;3;4}(\varphi^{*})$ . Here, a and b are the number of correct and incorrect diagnoses for carcinoma, and c and d are the same for adenoma. To estimate the levels of balanced accuracy Ac the gradation presented in Table 2.

**Table 2.** The balanced accuracy levels of differential diagnosis of benign and malignant prostate tissue samples.

Accuracy, Ac,%	Level	Colour marker
70%≤Ac≤80%	Satisfactory	
81%≤Ac≤90%	Good	
91%≤Ac≤100%	Excellent	

Table 3 summarises the mean and standard deviation of the sets of statistical moments. The results presented in Table 3 revealed the following levels of accuracy in the differential diagnosis of prostate tumors for a set of statistical markers:

**Table 3.** Statistical moments obtained for the optical anisotropy maps of prostate adenoma and carcinoma tissue samples and the corresponding balanced accuracy of cancer discrimination.

	Adenoma		Carcinoma		Accuracy, Ac,%	
$Z_{i}$	$\left\langle \Phi_{L}\right\rangle$	$\left\langle \Phi_{⊗,⊕}\right\rangle$	$\left\langle \Phi_{L}\right\rangle$	$\left\langle \Phi_{⊗,⊕}\right\rangle$	$\left\langle \Phi_{L}\right\rangle$	$\left\langle \Phi_{⊗,⊕}\right\rangle$
** $Z_{1}$ **	0.59±0.047	0.29±0.026	0.33±0.029	0.21±0.018	79	76
** $Z_{2}$ **	0.37±0.029	0.12±0.01	0.23±0.021	0.08±0.007	83	78
** $Z_{3}$ **	0.64±0.042	1.02±0.062	1.05±0.069	1.29±0.074	**91**	83
** $Z_{4}$ **	0.79±0.043	1.26±0.075	1.28±0.074	1.49±0.084	**92**	86
	** $\left\langle \Delta_{L}\right\rangle$ **	** $\left\langle \Delta_{⊗,⊕}\right\rangle$ **	** $\left\langle \Delta_{L}\right\rangle$ **	** $\left\langle \Delta_{⊗,⊕}\right\rangle$ **	** $\left\langle \Delta_{L}\right\rangle$ **	** $\left\langle \Delta_{⊗,⊕}\right\rangle$ **
** $Z_{1}$ **	0.12±0.009	0.065±0.0054	0.093±0.082	0.072±0.0067	73	72
** $Z_{2}$ **	0.14±0.012	0.083±0.0076	0.11±0.011	0.094±0.092	75	74
** $Z_{3}$ **	0.89±0.051	1.49±0.082	1.28±0.071	1.16±0.067	**93**	82
** $Z_{4}$ **	1.43±0.087	1.73±0.099	1.96±0.113	1.29±0.085	**94**	84

Maps of phase anisotropy $\left\langle \Phi_{L}\rangle (a\times b\right)$ and $\left\langle \Phi_{⊗,⊕}\rangle (a\times b\right)$ :

•
$Z_{1}$ – Satisfactory level Ac=76%−79% ;
•
$Z_{2}$ – Satisfactory $\left(\langle \Phi_{⊗,⊕}\rangle -78\%\right)$ and Good $\left(\langle \Phi_{L}\rangle -83\%\right)$ level;
•
$Z_{3}$ – Good $\left(\langle \Phi_{⊗,⊕}\rangle -83\%\right)$ and Excellent $\left(\langle \Phi_{L}\rangle -91\%\right)$ level;
•
$Z_{4}$ - Good $\left(\langle \Phi_{⊗,⊕}\rangle -86\%\right)$ and Excellent $\left(\langle \Phi_{L}\rangle -92\%\right)$ level.

Maps of amplitude anisotropy $\left\langle \Delta_{L}\rangle (a\times b\right)$ and $\left\langle \Delta_{⊗,⊕}\rangle (a\times b\right)$ :

•
$Z_{1}$ – Satisfactory level Ac=72%−73% ;
•
$Z_{2}$ – Satisfactory level Ac=74%−75% ;
•
$Z_{3}$ – Good $\left(\langle \Delta_{⊗,⊕}\rangle -82\%\right)$ and Excellent $\left(\langle \Delta_{L}\rangle -93\%\right)$ level;
•
$Z_{4}$ - Good $\left(\langle \Delta_{⊗,⊕}\rangle -84\%\right)$ and Excellent $\left(\langle \Delta_{L}\rangle -94\%\right)$ level.

The values obtained with the 3D MM reconstruction approach herein are compared with alternative techniques, including:

• polarisation mapping of the distribution of the azimuth α(x,y) of the polarisation of the sample [46,47,53];
• azimuthally-invariant polarisation mapping of the ellipticity β(x,y) distributions of the polarisation of the sample [46,47,53];
• 2D MM $\left(F_{ik}(x,y)\right)$ mapping of the sample [46,47];
• 3D MM reconstruction $\left(3D-\Phi_{L},\Phi_{C},\Delta_{L},\Delta_{C},\right)$ of the parameters of phase and amplitude anisotropy of the sample as described previously herein.

Two sets (16 samples each) of histological sections of biopsy of prostate adenoma and carcinoma with different levels of depolarisation (geometric thicknesses - l=(30μm;60μm;120μm)) were used in comparison with the alternative methods mentioned above. The results of the diagnostic effectiveness of four groups of polarimetry methods are presented in Table 4. Comparative analysis of the spatial polarisation mapping methods showed that for optically thin layers (Λ∼15%) the balanced accuracy of the 2D-based methods lies within a Good level, whereas 3D MM image reconstruction approach achieved an Excellent level. For the partially-depolarising layers (Λ≡25%−50%) the balanced accuracy of the approaches of spatial polarisation (α,β(x,y)) and 2D MM $\left(F_{ik}(x,y)\right)$ mappings substantially decreases from a Satisfactory level to an Unsatisfactory with an substantial increase of depolarisation (Λ≻25%−30%) .

**Table 4.** Balanced accuracy of various laser polarimetry methods for differentiating partially depolarising layers of benign and malignant prostate tissue samples.

	** α(a×b) **			** β(a×b) **		
** Λ,% **	∼15	∼45	∼85	∼15	∼45	∼85
** Ac,% **	75-80	55-65	50	80-85	60-65	50-55
	** $F_{ik}(a\times b)$ **			** $\left\langle \Phi_{L}\rangle ,\langle \Phi_{⊗,⊕}\rangle ,\langle \Delta_{L}\rangle ,\langle \Delta_{⊗,⊕}\right\rangle$ **		
	
** Λ,% **	∼15	∼55	∼85	∼15	∼45	∼85
** Ac,% **	90-95	70-75	65-70	**95-100**	90-95	65-70

Nevertheless, the 3D MM reconstruction approaches shows Good and Excellent levels of balanced accuracy. For strongly depolarising layers (λ≡55%−90%) all methods are ineffective. It is also clearly observed that introduced here 3D MM image reconstruction method gives superior diagnostic capabilities suitable for 2D methods extension.

## 4. Conclusion

We introduced the theoretical and experimental aspects of the 3D MM image reconstruction of layered distributions of linear and circular birefringence and dichroism of partially depolarising light of prostate benign and malignant tissue samples. The distributions of optical anisotropy were characterised statistically. Effective discrimination of prostate adenoma (τ=0.83;Λ=46%) and carcinoma (τ=0.81;Λ=45%) was achieved using the third and fourth order statistical moments of the linear dichroism and birefringence of the samples. A comparative analysis of the diagnostic efficiency of the existing 2D polarisation methods and the 3D MM image reconstruction of the optical anisotropy proposed herein showed its diagnostic superior across tissues samples of various thickness. This paves the way for a wider application of the proposed technology to the analysis and morphological imaging of optically-anisotropic polycrystalline structures and in particular to the differentiation of prostate tissue types.

## Data Availability

Data underlying the results presented in this paper are not publicly available at this time but may be obtained from the authors upon reasonable request.
